# Multicenter collaborative for orthopaedic research in India: An opportunity for global leadership

**DOI:** 10.4103/0019-5413.40252

**Published:** 2008

**Authors:** George Mathew, Parag Sancheti, Anil Jain, Mohit Bhandari

**Affiliations:** Division of Orthopaedic Surgery, McMaster University, Hamilton, Ontario, Canada; 1 Sancheti Institute for Orthopaedics and Rehabilitation, Pune, India; 2 Professor, University College of Medical Sciences and University of Delhi, Editor, Indian Journal of Orthopaedics

**Keywords:** Clinical trials, India, multicenter, research, trauma

## Abstract

Road traffic accidents are increasing at an alarming rate and have become a major public health concern in India. In addition, there is a lack of trauma research output and reliable data from India. There are several issues and challenges that have presented an opportunity for researchers and surgeons in India to develop a collaborative aimed at improving the quality and productivity of orthopaedic trauma research. Establishing a network of surgical researchers across India is a necessary first step towards global leadership in orthopaedic surgery trials.

Road-traffic accidents in India are increasing at an alarming annual rate of 3%, with a trauma related death occurring every 1.9 minutes. In 1997, 10.1% of all deaths in India were due to accidents and injuries and in 1998, nearly 80,000 lives were lost and 330,000 people were injured.^1^ Of these, 78% were men between the ages of 20-44 years, causing significant impact on productivity.^2^ A vehicular accident is reported every 3 minutes and a death every 10 minutes on Indian roads.^3^

Despite trauma being a major public-health problem, in India, no credible data is available to ascertain the outcome of trauma victims. A report by Peden *et al.*, emphasized the need for data on road traffic injuries, conditions of roads and vehicles and driver behaviour, as these are imperative for setting intervention priorities and formulating preventative policies.^4^ Currently, however, there is a lack of reliable data available from India. This is highlighted by a review of health research output from India for the year 2002 which revealed that of all the health research publications from India and those included in the PubMed database, only 0.8% reflected research in “injuries” (road traffic injuries: 0.1%), only a fraction of the 17% injuries that are known to cause ill-health burden in India.^5^

India is a country of contrasts with both extremes of affluence and poverty. It has the largest number of technologists and skilled scientists with state of art health services in metropolitan areas; however, it also has insufficient basic health services in rural areas.^6^ In contrast to North America, the natural history of disease is still available in India. By shear number India has adequate number of patients for any clinical problem so that efficient and valid research is possible in trauma and orthopedics. The health related needs of India are almost similar to the two third of world's population living in underdeveloped nations. The clinical research on late presentation of fractures, complicated polytrauma patients and late onset paraplegia following a sequalae of severe kyphosis of the healed tuberculosis spine is possible.

## ISSUES AND CHALLENGES

### Research and education

Trauma research output from India is currently insufficient and unfocussed and is therefore unable to inform the practice of medicine and surgery in the country.

Why? One plausible challenge is a lack of mentorship and education in good research practice and evidence-based orthopaedics. Researchers and medical students should be taught the principles and practice of clinical research and evidence-based medicine. In addition research ethics should form a significant part of their training. The enormous challenge of training quality professionals in clinical research can only be met by cooperative and collaborative efforts between industry, academia and government. Collaboratives with established researcher and education programs from around the world may also provide valuable research training within India.

The issues of educational standards, certification and continuing education and evaluation requirements for doctors involved in trauma care also need to be addressed. There are no minimum stipulated educational standards and formal education and specialty training in emergency medicine, trauma surgery and critical care are not mandatory for personnel involved in trauma care.^7^ The Indian Council for Medical Research (ICMR) have issued specific guidelines for international collaboration for research in biomedical sciences, which is a step towards better research practices.^8^

It is also necessary to bring about awareness and to educate the patient population in India. This will help mitigate problems regarding patient compliance issues. More effort is needed to create widespread awareness of clinical research amongst the general public, patients and the medical community.^9^

### Economic growth potential

Until 1990, India was not the preferred destination for major global pharmaceutical companies, although some of them were conducting clinical trials in India. During the last 10 years, however, India has seen an increasing global demand for world-class clinical trial management capacity and productivity. It is estimated that nearly 20% of all global clinical trials will be conducted in India by 2010.^10^ This increase is due to its rich technical resource pool, the relative ease and attractive economics of recruiting large numbers of patients and the sheer diversity inherent in the country's genetic structure.^11^

The commercial value of the Indian market for research and development (R and D) has a large growth potential. According to recent reports, clinical research outsourcing is perhaps, seeing the fastest growth. Pfizer has announced a doubling of its R&D spending in India, increasing their investment to approximately $13 million. They have increased their biostatistical and clinical trail logistics services in India 20-fold. Several other major pharmaceutical companies including Novartis, Astra Zeneca, Eli Lilly and GSK, are also committed to making India a global hub for their clinical research activities.^9^

The opportunities for orthopaedic research in India are staggering. With a biotechnology market of millions of dollars, the race to find novel therapies to improve fracture healing and quality of life in patients is a global priority. Indian orthopaedic surgeons must empower themselves with clinical research knowledge and research infrastructure to address this opportunity. Surgeons should not rely on outsourced expertise from Pharmaceutical companies, but rather develop their skills within India. Surgeons should lead and control the research within their specialities rather than risk being taken advantage of by multinational corporations. India should lead, not follow in global orthopaedic research.

### Capacity building

A lack of sufficient number of hospital sites meeting the International Council for Harmonization, Good Clinical Practice (ICH-GCP) norms is one of the main challenges the industry is facing in attracting large numbers of international clinical trials into India.^9^ Among some 14,000 general hospitals, no more than 150 have the adequate infrastructure to conduct trials and there are fewer than a dozen pathology laboratories that meet the criteria for compliance with good laboratory practice.^12^

While Pfizer has independently conducted more than 40 GCP workshops and trained more than 2,000 investigators in India, there is still a need at the present time for a centralized regulatory body which can guide high quality development of ethical capacity with extra vigilance and an informed understanding of the acceptable risks.^9^ Such a system, while conforming to international standards must also be uniquely Indian.

It is imperative to build the right kind of capacity to meet the anticipated demand for clinical trials in India. In as much as the optimism for growth in this industry, there is also an underlying concern that vulnerable populations may be exploited. New, sometimes difficult to meet expectations and balancing the need for local resources for basic health care are some of the immediate concerns. The regulatory regime in India is yet to identify ways of balancing the benefits and the risks.

There are currently no orthopaedic surgeon lead multicenter collaboratives in India that have been a successful model for the future. Similarly, industry sponsors are unfamiliar with orthopaedic surgical challenges and often inexperienced in developing the necessary collaborations to ensure successful fracture trials. Most, if not all the research training in India focuses upon drug trials and is led by industry. There are currently no organized and country-wide efforts to disseminate knowledge about orthopaedic research principles that relate to techniques or implants. To maintain pace with an ever-increasing demand for orthopaedic research, the approach to education has to be far more than a few GCP courses run by pharmaceutical companies. We need regional courses at all major orthopaedic meetings, earlier introduction of EBM principles and research during orthopaedic training and medical school and wide dissemination of home study materials (e-learning, self study courses).

### Bureaucratic obstacles

Obtaining regulatory approval in India is often a very slow process in comparison to Canada and the United Kingdom. This is because of inadequate funding and training of regulatory personnel.^9^ India's clinical research regulatory processes still need to be clearly defined. Although the Drugs Controller General of India is responsible for regulatory approvals of clinical trials in India, it is severely understaffed and lacks the expertise to evaluate protocols. This results in unnecessary delays in moving trial applications forward.^12^

### Data protection and intellectual property rights

Data collection involves considerable costs, time and energy and need to be protected as mandated in trade-related aspects of intellectual property rights (TRIPS) article 39.3.^13^ Recommendations are that India should allow at least 5 years data protection from the date of marketing approval.^9^

Another key issue that international organizations seek to resolve is India's position with regards to protection of intellectual property rights in accordance with international law. The representatives of India's knowledge-based industries, scientific research councils and the government are committed to respect and enforce the protection of intellectual property rights in accordance with international standards. The drug controller general has recently passed a ruling to enforce the exclusivity of clinical trial data.^14^

### Ethics and non-accredited clinical research organizations (CROs)

A key issue is related to ethics in research and clinical trials conduct in India, where regulatory and oversight environment are at an immature stage of development.^15^ Few of the large hospitals have institutional review boards that follow standard operating procedures and they, too, often lack the expertise with which to evaluate protocols. As a result, illegal and unethical trials have been conducted, several of which have attracted adverse coverage in the media in recent years.^12^

Another area of concern is the mushrooming of a number of new CROs without quality accreditation. There is no system of registration and/or approval of these organizations. Quality control and the potential for abuse remain a major concern. The government should ensure high standards through a system of monitoring by a regulatory agency.

Surgeons can improve the environment for research by ensuring proper training in clinical research principles, developing independent ethics boards to review research and protect patient safety and avoiding collaborations with contract research organizations that fail to provide sufficient proof of quality in their standards and conduct.

## THE NEED FOR MULTICENTER RESEARCH COLLABORATION IN INDIA

There are several advantages to a strategic alliance between orthopaedic surgeons, academia, the government and industry - the most important of which is the development of world-class expertise in the conduct of quality research and orthopaedic trials.

A partnership between public and private sector and with international organisations is a great way to develop and share expertise. Knowledge transfer from abroad and local expertise building should be according to established guidelines and efficiently coordinated. Quality control and joint-trials with reputed global players can give rise to building expertise in this area.

Establishing a research culture and conducting world-class trials is labour-intensive and process driven. The abundant and skilled manpower available in India could revolutionize the clinical trial field. India has to its advantage a large pool of motivated English-speaking scientists, about 3-4 million, the second largest concentration in the world following the United States.^16^ According to an industry report, India has about 500 investigators, over 572,000 doctors, 43,322 hospitals and dispensaries and approximately 807,000 beds including both private and public.^9^

The other benefits of participating in research and orthopaedic trials include:

- ***Access to Care***: The patient population having the opportunity to access cutting edge biomedical innovation, which could be life saving.
- ***Improved Health Outcomes***: There is substantial evidence that participation in research and clinical trials improves health outcomes.
- ***Financial and Material Gains***: Indian hospitals receive reimbursements for participating in clinical trials, either in funding, equipments and/or additional staff, which will benefit all patients served by that hospital.^16^
- ***Improved Research Skills***: Global clinical development programs are an opportunity for physicians and medical students to improve their research skills in accordance with international standards often leading to prized peer-reviewed publications.^9^
- ***Practice of Evidence Based Principles*:** A research environment will promote the clinical practice of evidence-based medicine.^16^
- ***Development of Regulatory and Monitoring Bodies***: Research collaboration with multinationals and global leaders would help the regulatory and oversight environment to quickly mature in keeping with ICH-GCP guidelines.^15^
- ***Medical Innovation***: Participating in clinical trials would enable physicians to develop the art of critical analysis and be on the cutting edge of new technologies and scientific innovation.^15^
- ***Economic Growth***: Clinical research creates economic opportunities and attracts talented professionals thereby creating jobs for site personnel, study monitors and ancillary services which impacts the whole local community.^15^

### Orthopaedic fracture trials: An unparalleled opportunity in India

**Figure 1:**
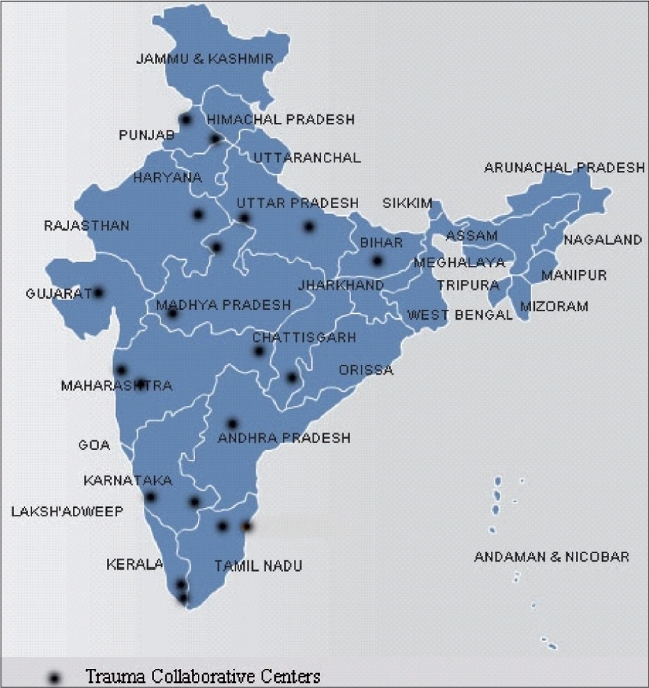
Indian multicenter orthopaedic research collaborative

We surveyed a number of orthopaedic surgeons in India to gain insight into their collaborative spirit and interest in a national multicenter research organization. Of 114 indian surgeons, 84 surgeons across 65 centers in India supported a research collaborative [Figure 1]. These centers have the potential to collectively recruit thousands of fracture patients per year for surgical trials [Table 1]. These numbers are magnitudes of order greater than similar recruitment capacity in North American Centers - the most popular location for orthopaedic research. The opportunity for global leadership is great in India.

**Table 1:** Capacity for yearly recruitment among Indian multicenter collaborative

Fracture type	Recruitment potential/ year multicenter collaborative (patients)
Closed tibial shaft fractures	8635
Open tibial shaft fractures	8455
Displaced femoral neck fractures	6340
Intertochanteric hip fractures	8184
Humerus fractures	6673

## CONCLUSION

Despite the challenges, India is well on its way to attracting high quality researchers and establishing itself as the global capital of surgical trials. The regulatory system is being strengthened and laws are being amended to facilitate the conduct of clinical trials. There is a focussed effort to increase training of research professionals thereby generating a large base of investigators and supporting staff. These initiatives will help India establish itself as a leader in global clinical research.

While cost-containment has been the driving force to the outsourcing of clinical trials, it is in the country's interest to quickly strengthen its position and change that focus to high quality and efficiency. Establishing a network of clinician researchers from India will help India fulfil its aspirations of becoming a major global player in orthopaedic trials.
